# Claims of the Microscope

**Published:** 1881-12

**Authors:** 


### CLAIMS OF THE MICROSCOPE.

It is true that microscopy is a science, and, perhaps, that truth deters many a young medical man from putting himself in a position to see nature and understand what he reads.

He entertains the idea that a microscope costs a fortune, and in order to understand it, he must possess an extraordinary genius, and devote all his time to it to be able to achieve anything in the line of microscopy—all of which is false, and we wish to remove, as far as possible, these erroneous and damaging impressions. Ordinary intelligence, with a determined purpose, and a few dollars, is what is needed.

Want of time is the excuse of many, and these men are too frequently found on a dry-goods box at the corner of the street, or some frequented place, either listening to, or telling, stories of questionable purity for hours together, and yet they have no time to spend with the microscope.

"Haven't the money to spare" is another cry we frequently hear. Watch and see if you do not misappropriate money enough annually for follies—for that which is not bread—for that which profiteth not. To be plain, we mean for drinks which becloud, blight, enfeble and ruin; also the accursed weed that contaminates, disgusts, and enslaves. Open your eyes. Take a pencil and make your own mathematical calculation, and see if you have not willingly spent more time and money for that which is worse than useless than the majority of first-class microscopists have in making themselves such.

We do not desire to enter the *morale* of the question, nor the finance, any further than to tear off the flimsy guaze, so that all may see for themselves whether it be a reality or a mere phantom. A word to the wise is sufficient. Cease to do evil; learn to do well. Save your money; economize your time; become a microscopist; strike boldly for the front of your profession.

*Western Medical Reporter.*

Poisonous cigars are continually being put upon the market, the poison in them being derived from disease and filth prevalent in the overcrowded tenament rooms in which they are manufactured.

